# Location-Optoelectronic Property Correlation in ZnO:Al Thin Film by RF Magnetron Sputtering and Its Photovoltaic Application

**DOI:** 10.3390/ma14216313

**Published:** 2021-10-22

**Authors:** Fang-I Lai, Jui-Fu Yang, Yu-Chao Hsu, Shou-Yi Kuo

**Affiliations:** 1 Department of Electrical Engineering Program C, Yuan-Ze University, 135 Yuan-Tung Road, Chung-Li 32003, Taiwan; filai@saturn.yzu.edu.tw (F.-I.L.); yoharecca@gmail.com (J.-F.Y.); 2 Department of Urology, Chang Gung Memorial Hospital, Linkou, No. 5, Fuxing Street, Kwei-Shan, Taoyuan 33305, Taiwan; hsuyc@cgmh.org.tw; 3 School of Medicine, Chang Gung University, 259 Wen-Hwa 1st Road, Kwei-Shan, Taoyuan 33302, Taiwan; 4 Department of Electronic Engineering, Chang Gung University, 259 Wen-Hwa 1st Road, Kwei-Shan, Taoyuan 33302, Taiwan

**Keywords:** CZTSe solar cell, AZO film, RF magnetron sputtering

## Abstract

In this study, a radio-frequency magnetron sputter system was used to deposit Al_2_O_3_ doped ZnO (AZO) thin films at room temperature, and the soda lime glass (SLG) substrates were placed at different zones relative to the center of the sample holder under the target. The samples were then analyzed using an X-ray diffractometer, Hall-effect measurement system, UV-visible spectrophotometer, and X-ray photoelectron spectroscopy. It was found that the electrical, structural, and optical properties of AZO films strongly depend on the target racetrack. The AZO thin film grown at a location outside the racetrack not only has the most suitable figure of merit for transparent conductive films, but also retains the least residual stress, which makes it the most suitable candidate for use as a CZTSe transparent conductive layer. When applied to CZTSe solar cells, the photoelectric efficiency is 3.56%.

## 1. Introduction

Cu_2_ZnSnSe_4_ (CZTSe) is a thin-film solar cell material, which has attracted much attention due to its high material absorption coefficient, abundant raw materials, and similarity in structure to Cu(In,Ga)Se_2_ solar cell components [1,2]. Currently, the common preparation methods of CZTS(Se) mainly include sputtering, co-evaporation, pulsed laser deposition (PLD), electroplating, and spin coating [3,4,5,6]. The hydrazine-based liquid process used by IBM has achieved a device efficiency of 12.7% [7]. However, hydrazine is toxic, prone to explosion, and requires expensive equipment. Therefore, it is impractical for use in mass production. During the past few years, CZTSe thin-film solar cells have demonstrated good performance in vacuum processes. The 11.4% efficiency of the sputtering-post-selenization method [4] and the 11.6% efficiency of the co-evaporation method [3] have proven to be the highest among the currently available vacuum methods, where the sputtering-post-selenization method has received the most attention. This method offers uniformity, flatness, a large area, and rapid mass production capabilities. The standard component structure of CZTSe thin-film solar cells consists of a transparent conductive layer (TCL), window, buffer, absorber, back contact, and substrate; the TCL is a key component. Within the wavelength range of 350–1400 nm, in addition to good electrical conductivity, the TCL must demonstrate high light penetration to reduce the absorption of incident light when it passes through it [8]. Many types of TCL are available [9,10,11], and In_2_O_3_:Sn (ITO) is the most commonly used in semiconductor components. The TCL commonly used in semiconductor components is In_2_O_3_:Sn (ITO). However, the In contained in the ITO material is a noble metal with a low output and is therefore, rather expensive [12]. In addition, panel factories currently still use ITO as the main TCL, implying that there are hidden concerns regarding the material supply chain of In. Moreover, ITO has several shortcomings, including poor chemical stability and potential harm to humans, as well as limitations when applied to flexible substrates [13]. Therefore, the development of low-cost In-free TCLs is very important.

Zinc oxide (ZnO) is an N-type semiconductor material with an energy gap of ~3.37 eV, which has a good transmittance in the visible light region. In addition to its abundant zinc content, low cost, good chemical stability, high transmittance, and stability in the near-infrared region, it can form a ZnO:Al_2_O_3_ (AZO) film through the doping of an appropriate amount of group III Al ions, which can then be used as a transparent conductive layer [14,15,16]. Therefore, AZO is considered a potential material for TCLs. The most commonly used methods for preparing AZO include sol-gel spin coating, atomic layer deposition (ALD), PLD, and magnetron sputtering [17,18,19]. The sol-gel spin coating method has yet to reach commercial standards in terms of conductivity, and the process requires annealing at high temperatures. In the later production stage of CZTS(Se) solar cells, such as in high-temperature environments, the efficiency of CZTS(Se) solar cells drops [20,21]. Although the ALD and PLD processes can produce good TCL characteristics, they are still not applicable when considering the aspects of production cost, large areas, and the commercialization of mass production. Therefore, most production lines in the industry currently use magnetron sputtering to deposit AZO thin films. It not only has the advantages of the sputtering method, such as low-temperature growth, large area, and easy-to-control process to produce good-quality films that grow easily [22], but also the magnetron technology to increase the plasma density of the target material, helping to increase the deposition rate, and improve the film stress and density, while minimizing the damage to the films. However, it causes significant plasma ion-bombardment in certain regions of the target material, causing uneven resistance on the surface of the target material, resulting in uneven spatial distribution. According to the literature, this affects the characteristics of the deposited film [23].

The characteristics of AZO film are affected by the parameters during sputtering, such as sputtering power, working pressure, distance from target to substrate, target specifications (Al doping concentration, density, purity, etc.), and substrate temperature during deposition. By adjusting the above parameters, high-quality AZO films can be obtained. However, most studies have pointed out that to deposit AZO films of good quality, the substrate needs to be heated to 200–400 °C during the deposition process [24,25]. However, if these high-temperature deposition conditions cannot be applied to polymer substrates and organic materials, the development and application of AZO will be limited. Therefore, the low-temperature preparation of high-quality transparent conductive AZO films is highly desirable. Still, only a few studies have reported the deposition of good-quality AZO films in low-temperature environments [11,26,27]. According to the literature, the lowest resistivity of an AZO film grown by sputtering at room temperature was 7.56 × 10^−4^ Ω cm, and the transmittance was 83% [11]. In addition, after the CZTSe solar cell is fabricated to the i-ZnO layer, for example, in high-temperature environments, the CdS layer may deteriorate, and the interface/structural properties of the device may be altered [20,21]. Therefore, high temperatures must be avoided during the production process. Therefore, low-temperature preparation of transparent conductive films is an important factor. This study focuses on the RF magnetron sputtering method for the deposition of AZO films in a low temperature environment and discusses the relationship between the substrate placed at different locations and the target etching area (i.e., the racetrack). The microstructure and photoelectric characteristics of the AZO films are measured by X-ray diffraction (XRD), X-ray photoelectron spectroscopy (XPS), a Hall-effect measurement system, ultraviolet-visual-near-infrared (UV-VIS-NIR) spectroscopy, and atomic force microscopy (AFM). The AZO film with the best figure of merit (FOM) which applies the least stress to the CZTSe solar cell is determined after calculation, with the optimum open circuit voltage (V_oc_) of 0.28 V, short-circuit current density of 28.29 mA/cm^2^, fill-factor of 0.45, and photoelectric conversion efficiency of 3.56%. In addition, the TCL layer prepared under a low temperature in this study is also applicable for organic photovoltaics, which must be maintained in a low-temperature environment during the production process [28,29].

## 2. Materials and Methods

**Figure 1:**
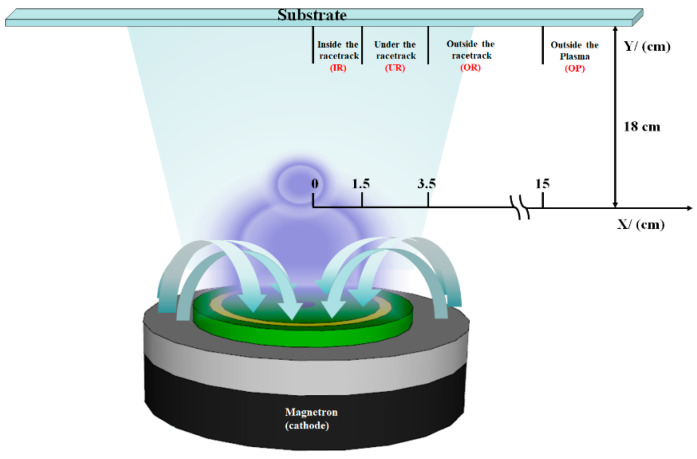
SLG substrates at different locations relative to target materials, divided into four regions: (1) inside the racetrack; (2) under the racetrack; (3) outside the racetrack; (4) outside the plasma.

In this study, the commonly used RF magnetron sputtering method was applied to grow AZO films. The target material used was a 3-inch commercial ZnO circular ceramic target (AZOY, purity 99.95%, density > 95%) (GfE, Nürnberg, Germany) doped with 2 wt% Al_2_O_3_, with a thickness of 3 mm. The AZO thin films were grown under pure argon (Ar, 5N) at a working pressure of 5 mTorr, on an unheated substrate with an RF power of 75 W. The thickness was fixed at ~300 nm. Since the relationship between the location of the substrate and the target is to be discussed, the substrates were arranged and fixed relative to the target position for deposition; the arrangement and sampling distances are shown in Figure 1. The substrates used were soda lime glass (SLG), and the locations of the substrates can be divided into four regions: (1) inside the racetrack (X < 1.5 cm), denoted as IR; (2) under the racetrack (1.5 cm < X < 3.5 cm), denoted as UR; (3) outside the racetrack (X > 3.5 cm), denoted as OR; and (4) outside the plasma (X > 3.5 cm), denoted as OP. The characteristics of the AZO films were identified using the SIEMENS D500 X-ray diffractometer (XRD) (SIEMENS, Texas, USA) with CuKα = 0.154 nm. The transmittance was determined with a UV-VIS NIR spectrophotometer (JASCO, MD, USA), using the SLG substrate as a reference, and the measured wavelength was between 300 and 2000 nm. A field emission scanning electron microscope was used to observe the morphology and thickness of the film, while the sheet resistance (R_s_) was obtained using the Hall measurement. The best deposition parameters of the AZO thin films were thus obtained and used in CZTSe solar cells.

The production process of CZTSe solar cells was as follows: a layer of Mo was deposited as the back electrode on the SLG substrate using the sputtering method, Zn_x_Sn_1−x_ and Cu_x_Se thin films were deposited as the precursors by sputtering as well. The thicknesses of Zn_x_Sn_1−x_ and Cu_x_Se layers were 500 nm and 250 nm. During the precursor deposition process, the rotation speed of the carrier disk was maintained at 15 rpm. The thickness of Zn_x_Sn_1−x_ and Cu_x_Se layers was 500 nm, and 250 nm. The deposited precursor test piece was placed in a quartz box together with 0.7 g of Se ingot, and Ar was passed to allow for selenization (maximum temperature of 550 °C). After selenization, the thickness of the CZTSe film was approximately 1 μm. After depositing a 150 nm thick buffer layer of CdS and a 50 nm thick layer of i-ZnO by the sputtering method, the AZO film was deposited using the optimum deposition conditions obtained in this study. Finally, an interdigitated electrode grid of Ni and Al was deposited to complete the entire CZTSe thin film solar cell. The prepared CZTSe solar cell was tested for the current density–voltage (J–V) characteristics and external quantum efficiency (EQE).

## 3. Results and Discussion

**Figure 2:**
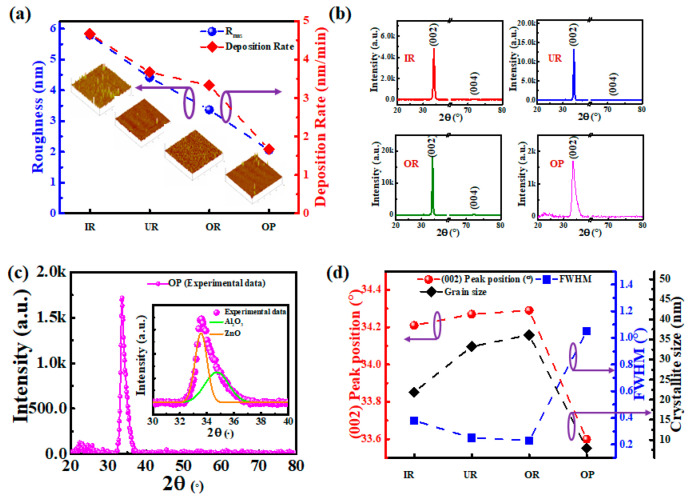
SLG substrates at different locations relative to target material, with the deposited AZO film characteristics as follows: (**a**) deposition rate and AFM measurements; (**b**) XRD measurements; (**c**) XRD magnified fitting results at location OP; (**d**) position shift of (002) plane main peak and calculations of FWHM and crystallite size.

Figure 2a shows the deposition rate and surface roughness of the AZO films grown on the SLG substrates at different locations relative to the target. Under the same parameters, the deposition rates of the deposited AZO films are 4.67 nm/min for IR, 3.67 nm/min for UR, 3.33 nm/min for OR, and 1.67 nm/min for OP. This shows that the farther away from the IR position, the lower the film deposition rate, which means that the deposition energy also shows the same trend. The surface roughness of the AZO films grown on the SLG substrates at different locations relative to the target measured by AFM is such that IR = 5.79 nm, UR = 2.27 nm, OR = 2.12 nm, and OP = 1.64 nm, respectively. This is because the faster the deposition rate, the more the deposition energy is generated. In addition, the faster the deposition rate, the faster the film grows, resulting in a larger surface roughness. Figure 2b shows the XRD measurement results of the AZO thin films grown on the SLG substrates at different locations relative to the target. All the AZO films have an obvious peak along the ZnO (002) plane, which means that the film has a wurtzite (hexagonal) structure where the c-axis is preferentially grown in the direction perpendicular to the substrate surface. In addition, the intensity of the main peak of the (002) plane is the strongest in OR, followed by that in UR, IR, and OP, and the change in intensity is related to the film quality and the size of the crystal grains [12]. Moreover, the AZO film grown at the OP location has an obvious asymmetric peak on the (002) plane. Figure 2c shows the XRD pattern of the AZO film deposited in the OP region. The inset is a zoomed-in view of the 30–40° region. From Gaussian fitting, it is estimated that the primary contributions of diffraction peak planes are from ZnO and Al_2_O_3_. This phenomenon is likely caused by a too low a deposition energy, which makes the quality of the ZnO poor, and Al^3+^ failing to obtain sufficient energy to replace Zn^2+^, such that it remains in the form of the Al_2_O_3_ compound. Figure 2d is a graph showing the changes of full width at half maximum (FWHM) and the position of the main peak of the (002) plane, as well as the crystallite size calculated by Scherrer’s formula. First, the main peak of the undoped ZnO (002) plane is ~34.4167° (JCPDS No. 36-1451). Among the four deposition locations in this study, the main peak of OR is closest to that of undoped ZnO, followed by UR, IR, and OP. The main reason for the displacement of the (002) main peak is stress, which is mainly caused by extrinsic and intrinsic factors that cause lattice deformation. The extrinsic factors are primarily related to either the lattice constant, thermal expansion coefficients (TEC) between the film and the substrate, or both. The TEC of the ZnO and SLG substrates used in this study are approximately 4.75 × 10^−6^ K^−1^ and 8.6 × 10^−6^ K^−1^, respectively [15]. Except for the small difference in TEC, this study realizes the deposition on unheated substrates and employs SLG substrates, which are an amorphous material. Therefore, the stress caused by extrinsic factors can be ignored. The stress caused by intrinsic factors mainly occurs during the growth of the film, such as heterogeneous plastic deformation, crystallite coalescence, defect formation, phase transformation, and energetic particle bombardment. Depending on the material and manufacturing process, some or all of these factors can cause different degrees of tensile or compressive strain. The in-plane stress ($\sigma_{\mathrm{film}}$) in the AZO film is estimated from XRD measurements using the biaxial stress model [30]. For a hexagonal lattice:

(1) $$\sigma_{\mathrm{film}}^{\mathrm{XRD}}=\frac{2c_{13}^{2}-c_{33}\;\left(c_{11}+c_{12}\right)}{2c_{13}}\cdot \frac{c_{\mathrm{film}-}c_{\mathrm{bulk}}}{c_{\mathrm{bulk}}},$$

where $c_{\mathrm{ij}}$ is the elastic stiffness tensor of a single ZnO crystal, $c_{11}$= 208.8, $c_{12}$= 119.7, $c_{13}$= 104.2, $c_{33}$= 213.8 GPa, and $c_{\mathrm{bulk}}$ is ~5.206 Å [31]. $c_{\mathrm{film}}$ is used to calculate the plane spacing of the hexagonal lattice (*hkl*) with the following formula:

(2) $$d_{hkl}={\left[\frac{4\left(h^{2}+hk+k^{2}\right)}{3a^{2}}+\frac{l^{2}}{c^{2}}\right]}^{-\frac{1}{2}}$$

The lattice constants calculated based on the c-axis (002) plane main peaks are respectively 5.236 Å for IR, 5.227 Å for UR, 5.224 Å for OR, and 5.328 Å for OP. The competitive stresses calculated by the *c*-lattice constants are shown in Table 1, where OR has the lowest value. 

From a mechanical point of view, the position of the main peak of (002) is shifted from that of the standard (bulk), and the representative samples show macro residual stress in the film. In the field of micro-electronic devices, stress affects not only the mechanical stability (adhesion) of the film structure, but also the electronic properties of the device. According to a study on CuInSe_2_ (CIS) solar cells, when the stress between ZnO and the window layer is too large, the band offsets are adversely affected, and the efficiency of the cell is reduced [16]. Since CZTSe and CIS have the same type of solar cell stack structure, it is believed that CZTSe solar cells will show the same results. Therefore, OR has the most suitable characteristics for the CZTSe solar cells. In terms of FWHM, OR has the best FWHM value, followed by UR, IR, and finally OP. This phenomenon is attributed to the influence of the energy in the plasma during deposition. If the deposition energy is too small, the crystalline material will not grow well, but if the energy is too large, it will bombard the film and cause damage to the material [15]; the stoichiometric ratio in the film also affects the structure changes.

**Figure 3:**
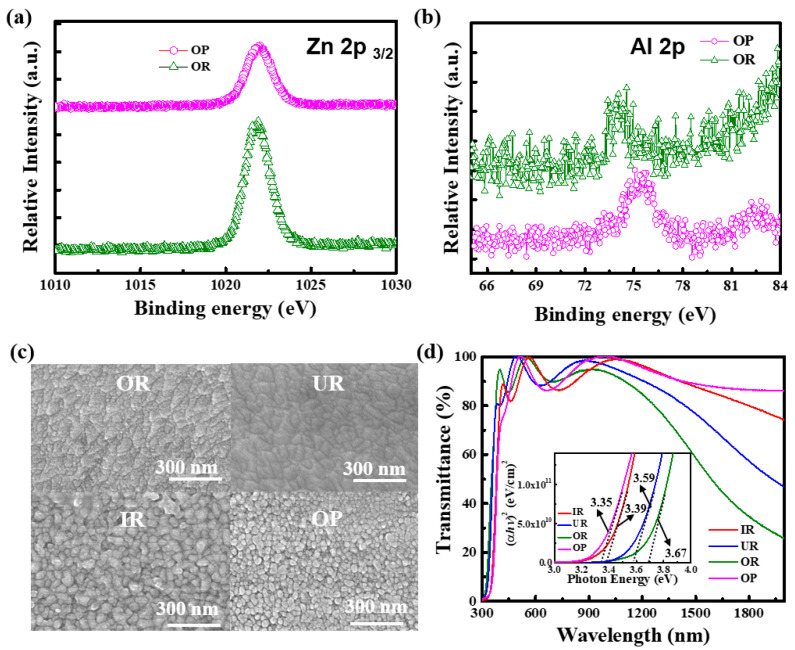
SLG substrates at different locations relative to target material, with deposited AZO film characteristics as follows: (**a**) XPS spectra of Zn 2p_3/2_ at locations OP and OR; (**b**) Al 2p binding energy measurements; (**c**) SEM measurements; (**d**) optical transmittance.

To further verify the results of the XRD, XPS analysis was performed for the Zn 2p_3/2_ and Al 2p of the two AZO films with the largest differences from locations OR and OP, as shown in Figure 3a,b. The binding energy of Zn 2p_3/2_ shows a typical ZnO matrix at 1021.9 eV, which confirms that most of the Zn atoms maintain the same formal valence as Zn^2+^ in the ZnO matrix. The Al 2p spectra are successfully detected despite the low Al concentration of the AZO films at the OR and OP locations, where the binding energy is 74 eV at OR, with a representative ZnO lattice showing that Al^3+^ replaced the Zn^2+^ sites, indicating that the Al doping is successful [32]. The binding energy of the AZO film at the OP location is 75.3 eV, which is the same as that of Al_2_O_3_, showing that Al_2_O_3_ is formed and possibly exists at grain boundaries [33], thus confirming the XRD results. Figure 3c shows the SEM top views of the AZO films deposited at the locations of IR, UR, OR, and OP. It can be seen that the surfaces of the films deposited in the OR and UR regions are continuous and dense. Film surfaces in the IR and OP regions appear granular. In the IR region, the particles on the film surface are larger and more uniform than those in the OP region. This is because, in the OR and UR deposition regions, the proper atomic deposition kinetic energy and deposition rate are conducive to the grain coalescence phenomenon. This allows sufficient time for atom arrangement, which leads to the formation of a more continuous and denser film surface. In the IR deposition region, however, because of the high atomic deposition kinetic energy and rapid deposition rate, the atoms have insufficient time for proper arrangement, which in turn limits the grains and increases the surface roughness. By contrast, in the OP deposition region, the low atomic deposition kinetic energy is insufficient to move atoms to appropriate positions, which leads to the formation of a surface with relatively small granular particles. Table 1 shows the measurement results of the sheet resistance of the AZO films grown on the SLG substrates at different deposition regions. It is found that for IR, UR, OR, and OP, respectively, the resistance is 1.22 × 10^−2^, 2.08 × 10^−3^, 3.17 × 10^−4^, and 18.9 Ω cm; the carrier concentration is 8.49 × 10^19^, 6.84 × 10^20^, 1.11 × 10^21^, and 2.85 × 10^15^ cm^−3^; and the carrier mobility is 2.07, 4.4, 6.13, and 116 cm^2^/V·s. The AZO film at OR has the optimum resistance, while the film at OP has the optimum carrier mobility. The main reason that affects electrical properties may be attributed to the AZO crystallinity influenced by deposition energy.

Figure 3d shows the measurement results of the transmittance of the AZO films grown on the SLG substrates at different locations relative to the target (wavelength ranging from 300 to 2000 nm). The average transmittance in the visible light region (400–800 nm) is also listed in Table 1, and the average transmittance is approaching ~90%. However, significant changes have taken place in the NIR zone, such that the penetration rates of UR, IR, and OP are higher than that of OR. The reason for this phenomenon can be attributed to the excessively high carrier concentration leading to free carrier absorption. In addition, the inset is an optical band gap energy (E_g_) shifted in the range from 3.35 to 3.67 eV in Figure 3d. In a direct-bandgap semiconductor system, the bandgap energy of an AZO film was calculated from the absorption coefficient by extrapolating the linear region in the plot of ${\left(\alpha hv\right)}^{2}\;$versus photon energy (hv) and by taking the intercept on the hv-axis according to the following relationship [34]:

(3) $${\left(\alpha hv\right)}^{2}=hv-E_{g},$$

where h is Plank’s constant and v is the photon frequency. According to the transmittance spectrum, the energy gaps are calculated to be IR ~3.39 eV, UR ~3.59 eV, OR ~3.67 eV, and OP ~3.35 eV. The increase in carrier concentration causes the Burstein–Moss effect, which leads to the blue shift of the AZO film absorption edge. A higher carrier concentration indicates that more Al^3+^ has been incorporated into the ZnO lattice and has substituted the Zn^2+^ sites, thus leading to an increase in the energy gap. However, the OP deposition region is dominated by ZnO and is only affected by a small amount of Al_2_O_3_. Therefore, the change is not significant. FOM (Φ_TC_) is one of the important bases for evaluating TCL, which is defined as Φ_TC_ = T^10^/R_S_, where T is the average transmittance of visible light (400–800 nm), and R_s_ is the sheet resistance.

**Figure 4:**
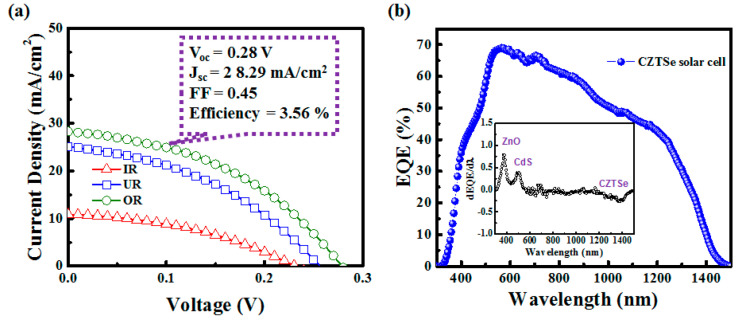
(**a**) J–V measurements for CZTSe solar cells prepared using IR, UR, and OR parameters; (**b**) EQE measurement for the CZTSe solar cell prepared using the OR parameter.

The Φ_TC_ of the AZO films grown on the glass substrates at different locations relative to the target is also listed in Table 1. The calculated Φ_TC_ is the largest for OR at ~4.35 × 10^−2^, which is a rather good value in literature related to AZO TCL [35,36,37]. OR also has the smallest stress, making it the most suitable for TCL application, followed by UR and IR. Figure 4a shows that after the i-ZnO/CdS/CZTSe/Mo/SLG layers were prepared (see the manufacturing method described in the experimental procedure), the component was placed in the IR, UR, OR, and OP regions for AZO film deposition. The Al/Ni interdigitated electrode grid was then deposited to complete the CZTSe solar cells. The J–V diagrams were measured under the AM 1.5 G condition. The conversion efficiencies were IR at 0.99%, UR at 2.57%, OR at 3.56%, and OP at 0%. In general, the changes in open-circuit voltage and short-circuit current density are primarily determined by the conductivity of the AZO film. This is because the TCL layer has a low conductivity and thus reduces electron transfer, lowering the probability of electrons being collected by the Al electrode. This decreases the current density and in turn affects the open-circuit voltage and efficiency. The trend of efficiency change is consistent with that of FOM. Note that the CZTSe solar cell deposited with AZO film in the OR region exhibited the highest conversion efficiency of approximately 3.56%. Figure 4b is an EQE diagram of the CZTSe solar cell with an AZO film deposited in the OR region. The wavelength is in the range of 300–1500 nm, and the maximum quantum response of approximately 70% is observed at approximately 565 nm. In addition, the inset shows the differential of EQE with respect to wavelength. It can be found that the absorption edges of ZnO, CdS, and CZTSe at 370 nm, 520 nm, and 1400 nm are similar to those recorded in the literature [21].

## 4. Conclusions

In this study: the optical, electrical, and microstructural properties, as well as the morphology of the AZO films corresponding to different regions of the target material were investigated and discussed. Under the same conditions, an AZO film with a resistivity range from 18.9 to 3.17 × 10^−4^ Ω cm and an average visible light transmittance of ~90% was obtained and was employed in CZTSe solar cells with a conversion efficiency of ~3.56%. Although the range of resistivity varies greatly, if it can be effectively controlled to achieve the simultaneous selection of deposition positions according to the characteristics required for various components to meet their needs, the cost can be reduced effectively.

## Figures and Tables

**Table 1:** Characteristics of AZO film in different deposition regions and the corresponding efficiencies when applied to CZTSe solar cells.

Sample Location	IR	UR	OR	OP
p (Ω cm)	1.22 × 10^−2^	2.08 × 10^−3^	3.17 × 10^−4^	18.9
n (cm^−3^)	8.49 × 10^19^	6.84 × 10^20^	1.11 × 10^21^	2.85 × 10^15^
µ (cm^2^/V·s)	2.07	4.4	6.13	116
R_s_ (Ω)	407.6	69.2	10.57	6.3 × 10^5^
T (%)	90.01	92.22	92.52	89.59
Φ_TC_	8.56 × 10^−4^	6.43 × 10^−3^	4.35 × 10^−2^	5.29 × 10^−7^
$\sigma_{\mathrm{film}}^{\mathrm{XRD}}\;$(Gpa)	0.23	0.16	0.14	N.A.
Efficiency (%)	0.99	2.57	3.56	N.A.

## Data Availability

The data presented in this study are available upon request from the corresponding author.
